# Effects of Rapid Weight Loss on Kidney Function in Combat Sport Athletes

**DOI:** 10.3390/medicina57060551

**Published:** 2021-05-31

**Authors:** Nemanja Lakicevic, Antonio Paoli, Roberto Roklicer, Tatjana Trivic, Darinka Korovljev, Sergej M. Ostojic, Patrizia Proia, Antonino Bianco, Patrik Drid

**Affiliations:** 1 Sport and Exercise Sciences Research Unit, University of Palermo, 90133 Palermo, Italy; antonino.bianco@unipa.it; 2 Department of Biomedical Sciences, University of Padova, 35122 Padova, Italy; antonio.paoli@unipd.it; 3 Faculty of Sport and Physical Education, University of Novi Sad, 21000 Novi Sad, Serbia; roklicer.r@gmail.com (R.R.); ttrivic@yahoo.com (T.T.); korovljev.darinka@gmail.com (D.K.); sergej.ostojic@chess.edu.rs (S.M.O.); patrizia.proia@unipa.it (P.P.); patrikdrid@gmail.com (P.D.)

**Keywords:** weight cutting, making weight, creatinine, blood urea nitrogen, urine specific gravity, renal system, health, performance

## Abstract

Even though scientific literature shows numerous heath complications and performance decrements associated with rapid weight loss (RWL), its prevalence remains exceedingly high across various combat sports. The aim of this study was to thoroughly search the existing literature to explore the influence of RWL on kidney function in Olympic and non-Olympic combat sport athletes. PubMed and Web of Science were searched for the relevant studies. Only original articles published from 2005 onwards, written in English, that included healthy males and females who prompted ~5% weight loss within a week or less, were included in the study. Retrieved studies showed that creatinine, blood urea nitrogen and urine specific gravity values were significantly increased after RWL in the majority of the included studies. This observation indicates that RWL caused dehydration and subsequent acute kidney damage despite various degrees of weight lost during the RWL phase, which can lead to adverse events in other body systems. Alternative methods of weight reduction that prioritize athletes’ health should be considered.

## 1. Introduction

The weight division rules in combat sports have been established in order to give every athlete a fair chance to compete in his/her given category. In principle, athletes of the same anthropometric properties have similar physical abilities and, as such, are suitable to compete in their predetermined weight class [1]. However, combat sports athletes regularly engage in rapid weight loss (RWL) so they can compete in the upper spectrum of a lower-weight category and presumably gain the competitive advantage over their lighter opponents. However, several studies deny such assumptions and indicate that RWL is not necessarily associated with competitive success [2] or good performance [3,4], making the RWL practice controversial. The phenomenon of RWL is omnipresent in combat sports [5], but varies in terms of its prevalence and percentage of weight lost prior to competition. Methods to induce RWL are largely similar between combat sports and commonly include reduced fluid and food intake, sauna use, training in heated rooms and rubber suits, etc. [6]. The synergistic effect of food restriction and fluid deprivation creates a “perfect storm” for adverse physiologic effects on the body, leaving the athlete ill-prepared for the competition [7]. It seems that RWL is routinely conducted based on anecdotal evidence that is rooted in dangerous practices that can have serious consequences [8]. Moreover, some combat sport athletes have reported repeating the RWL process up to 10 times per year [9]. Over the course of years, existing literature has thoroughly explained the health consequences associated with RWL [1,5,10], which range from acute to chronic and can even lead to lethal outcomes [11]. Despite a growing body of evidence admonishing this type of behavior, athletes still persist in this practice with prevalence of RWL reaching 90% in some combat sports [9].

As nearly 65% of the human body is made of water, which makes it a good source of significant and temporary RWL [12], intentional dehydration is the culprit of RWL [7] and thus it seems reasonable to expect its great influence over kidney function (renal system). To date, a considerable number of studies have been conducted on the effects of acute dehydration in combat sports, both solely and within RWL, but to our knowledge, no comprehensive review has been published on the impact of RWL on kidney function. Therefore, the aim of this study was to thoroughly review the literature to determine the effects of RWL on kidney function in combat sport athletes.

## 2. Materials and Methods

### 2.1. Search Strategy

EndNote software (Clarivate Analytics, Jersey, UK, ver. 9.1.) was used for the organization of content obtained by the article searches. Web of Science and PubMed were two electronic databases explored for the article acquisition. The following string was applied: “rapid weight loss” AND “kidneys”; “rapid weight loss” AND “kidney function”; “rapid weight loss” AND “renal system”; “rapid weight loss“ AND “renal function”; “making weight” AND kidneys; “making weight” AND “kidney function”; “making weight” AND “renal system”; “making weight” AND “renal function”; “weight-cutting” AND kidneys; “weight-cutting” AND “kidney function”; “weight-cutting” AND “renal system”; “weight-cutting” AND “renal function”. Once the search was completed, article screening was carried out in a three-step procedure: title reading, abstract reading and finally full-text reading. If any disputes were shown between the two investigators, a third member of the research team analyzed the current process independently and discussed the decision with the other investigators. Notably, investigators were not blinded to the manuscripts, study title, authors or associated institutions during the selection process.

### 2.2. Inclusion and Exclusion Criteria

Only original articles written in English and published in peer-reviewed journals were considered for inclusion within this review. Combat sport athletes (Olympic and non-Olympic sports) had to engage in RWL which prompted ~5% weight loss within seven days or less. However, if a certain study had athletes engaging in RWL that lasted longer but induced a higher percentage body weight lost, it was still included in the analysis. The date limit for publication period was set from the year 2005 until February 2021. Various formats of publications, such as reviews, meta-analyses, abstracts, citations, scientific conference abstracts, opinion pieces, books, book reviews, statements, letters, editorials, non-peer reviewed journal articles and commentaries, were excluded. There was no age limit for the participants and both genders were eligible to be included in the review. If a particular study reported illegal substance use to elevate the magnitude of RWL, it was still included in the study for further analysis.

### 2.3. Data Extraction

Essential information about included studies was delineated through tables (Microsoft Word 2016, Microsoft, Washington, DC, USA) while a narrative description was adopted to depict certain specifics about a particular study that expanded beyond tabular explanation. Retrieved data acquired from included articles dealt with the influence of RWL on biomarkers of kidney function.

### 2.4. Risk of Bias Assessment

The Physiotherapy Evidence Database (PEDro) scale was used for risk of bias assessment [13]. The PEDro checklist contains questions regarding eligibility criteria, sample randomization and blinding of the subjects and researchers. The maximum score a study could receive is 11, with higher scores denoting greater quality. Two independent scholars completed the PEDro scale for the included articles to determine the quality of each individual study. The inter-rater reliability (IRR) statistical method was conducted in SPSS (IBM, New York, NY, USA, v.20) to determine the level of agreement between two scholars with reference to scoring of included studies.

## 3. Results

A preliminary title and abstract search yielded 726 hits. Due to lack of relevance or inadequate study design, only three studies found through systematic search met our inclusion criteria. Initially, this judgement was made solely based on the title and the abstract and was later confirmed after full-text reading. In addition, a senior researcher (full professor) suggested another seven studies which did not include keywords used in the initial search, but were found by searching bibliographies of other studies, and met our inclusion criteria with respect to study design. Therefore, a total of 10 studies was included in this review. The retrieved studies were comprised of athletes training Muay Thai [14], wrestling [15,16,17], Taekwondo [18], mixed martial arts [19,20,21] and judo [22], which included a total of 171 participants (Table 1). After article acquisition, we identified creatinine (Cr) and blood urea nitrogen (BUN) as the most commonly detected biomarkers used to determine the influence of RWL on kidney function, while urine specific gravity (USG) indicated the level of dehydration of combat sport athletes. The average PEDro risk of bias score was 5 and IRR was 0.80, which can be considered fairly well.

In a study by Cannataro et al. [14], the authors recruited 21 high-level Muay Thai fighters (m = 13, f = 8; 25.8 ± 2.52 years; 68.03 ± 11.56 kg; 1.71 ± 0.08 m) to examine the effects of supervised RWL and rapid weight gain on various biomarkers, hormones and body composition. However, for the purpose of our study we focused only on RWL. The RWL lasted 3 days and was primarily induced by severe caloric restriction (−1000 kcal per day) whereby less than 30 g of carbohydrates per day was ingested, while 2 g of protein per kg of body mass per day and 0.5 g of fats per kg of body mass per day were consumed. The main emphasis was placed on glycogen depletion from muscle and liver deposits, which would result in significant loss of body water. In addition, as a part of supervised RWL, all participants received dietary micronutrient and phytochemical supplements daily. Concurrently, athletes engaged in <2 h of low-intensity endurance exercise (approximately 60% of VO2max), which was far from their usual vigorous-intensity training regimens. Significant changes in intracellular and extracellular water (*p* < 0.05) were noticed in females after RWL, whereas their male counterparts did not experience significant change. On average, athletes lost 4.1% of their initial body weight, which resulted in significantly increased BUN (23.7 ± 3.4 mg/dL) in all athletes, with a marginal increase in Cr values in males (1.03 ± 0.09 mg/dL) and no changes in females (0.93 ± 0.03 mg/dL).

Eighteen elite wrestlers (21.9 years, range 17.8–31.7 years) participated in a study by Karila et al. [15] where the authors examined the impact of RWL on body composition fluctuations and blood chemistry. Over a period of two to three weeks, athletes had to restrict their caloric intake mainly by decreasing carbohydrate and fat intake, while protein intake was advised to be 2 g per kilogram daily. During initial RWL, energy intake was restricted to 800–2000 kcal per day depending on the daily energy expenditure and the amount of weight loss. However, final weight cut was achieved over the last two days mainly by perspiration (heavy exercise in a hot sauna) whereby extreme dehydration was applied and caloric intake was 500–1000 kcal daily (including electrolyte solutions provided). Average body weight loss after RWL was 5.7 ± 1.5 kg (8.2 ± 2.3%), while maximal weight loss in a single subject was 8.3 kg (11%). Significant reductions in fat tissue (0.9 ± 0.4 kg (16 ± 6.9%)) and muscle tissue (4.8 ± 1.3 kg (7.9 ± 2.5 %)) were found. Following RWL, serum Cr values reached 120 ± 25 μmol/L and there was a significant correlation between RWL and Cr values (*p* ≤ 0.001). Additionally, there was a significant correlation between reduced lean body mass and increased serum Cr (*p* ≤ 0.001).

Twenty-four Turkish national team wrestlers (19.33 ± 0.70 years, 173.00 ± 8.26 cm) participated in a study by Cicioglu et al. [16]. The authors elaborated on the pre-competition weight loss patterns and found that 62.5% of the participants lost weight whereas 37.5% did not. Among those engaging in weight loss, 60% of them prompted RWL 1-7 days prior to competition whereas 40% of them performed RWL 8-14 days before the competition. Methods of weight loss were not revealed in the study. Results showed that RWL group reduced −5.73% of their body weight, while reductions of −11.91% and −4.27%, were detected for fat mass and fat free mass, respectively. Of concern, a decrease of −5.30% in total body water was noted in RWL group. In contrast, non-weight loss group did not show statistically significant differences in terms of body mass index and fat mass while statistically significant differences occurred with respect to fat free mass and total body water (*p* < 0.05). In RWL group, pre to post analysis showed significant increase in BUN (17.31 ± 3.68 mg/dL; *p* < 0.01), while no statistically significant changes were noted in the non-weight loss group. 

A study by Ozkan and Cicioglu [23] on 69 wrestlers (22.51 ± 2.49 years, 174.54 ± 6.59 cm, 78.98 ± 15.87 kg) examined the impact of the RWL phase prior to the Turkish Wrestling Championship. A special emphasis was placed on the effects of dehydration during a 1–7-day time-frame. The results showed that 55% of athletes underwent RWL whereby 4.55 ± 1.87% of body weight was lost. The group that was voluntarily subjected to RWL showed significant increases in BUN when compared to group that did not lose weight (*p* < 0.05).

In a study by Yang et al. [18], 10 taekwondo athletes (21.1 ± 5.48 years, 1.74 ± 0.08 m, 71.6 ± 11.1 kg) were subjected to either RWL that included 5% weight loss achieved over 4 days or gradual weight loss (GWL) that was achieved over 4 weeks. During RWL, weight reduction was attained via individually used methods which included elevated training intensity and training sessions with thermal clothing, fasting and dehydration. Conversely, the GWL group achieved weight loss primarily by combining exercise with minimal caloric intake. However, all athletes continued their normal training schedule (6–8 h per week) with an additional running training session (1 h/week). Creatinine values were unchanged in the GWL group while significant increases were detected in the RWL group (1.12 ± 0.03; *p* = 0.045).

Sixteen wrestlers (22.5 ± 3.9 years; 79.4 ± 7.2 cm; 81.4 ± 9.7 kg) participated in a double-blind placebo study by Timpmann et al. [17]. The participants had to reduce 5% of their weight during three days. Sixteen hours after RWL (self-selected methods), all of the subjects consumed water ad libitum and ate an identical supervised diet. The diet was supplemented with wheat flour as placebo for eight wrestlers and with sodium citrate (buffering agent) for the other eight wrestlers. Urine specific gravity (USG) was used as an indicator of hydration status. The retrieved data of the two groups showed a significant increase in USG during RWL (from 1.0176 ± 0.0041 to 1.0290 ± 0.0028, *p* < 0.001). However, USG returned to normal values during the 16 h recovery, and no between-group differences existed in USG at any stage of the study.

Eight judo athletes (19.3 ± 2.0 years, 178.1 ± 6.3 cm, 81.7 ± 10.7 kg) took part in a study by Drid et al. [22] where authors evaluated the effects of a 7-day RWL (restriction of fluid and food intake) on body composition and biomarkers of creatine metabolism during a pre-competition period. Still, athletes were prescribed vitamins and electrolyte supplements containing no calories. The volunteers were assessed on two occasions separated by seven days. Serum Cr levels were significantly increased at follow-up (mean difference 10.9 μmol/L, *p* = 0.009; 95% CI 0.2 to 22.0 μmol/L), while serum Cr was not affected during the study (*p* > 0.05).

Forty MMA fighters (m = 38; f = 2; 25.2 ± 0.7 years; 1.77 ± 0.01 m; 75.8 ± 1.5 kg) were included in a study by Jetton et al. [20] who aimed to quantify the extent of dehydration as an essential component of RWL prior to an MMA event. A significant increase (*p* < 0.001) was found for USG from the official weigh-in to 2 h before competition. The USG decreased from 1.028 ± 0.001 to 1.020 ± 0.001. Furthermore, 39% of the subjects were significantly dehydrated (USG > 1.021), and of those subjects 11% were seriously dehydrated (USG > 1.030) when assessed 2 h before competition. A matter of concern is that only 23% of the subjects were classified as “well hydrated” (USG < 1.010) in the 2-h period before competition.

Kasper et al. [21] monitored an elite male MMA athlete (22 years; 80.2 kg; 1.80 m) during a two-month training period and obtained regular assessments of kidney function throughout the study. The athlete was required to make weight at 65.7 kg from 80.2 kg over an 8-week period. The athlete underwent several phases in order to reduce weight rapidly, but overall followed a “low carbohydrate” diet for the duration of the entire study. During the first phase (−8 until −4 weeks), the athlete reported a daily caloric intake from 1500–1900 kcal (1.5–2.5 g/kg CHO, 1.6–1.8 g/kg protein, 0.8–1 g/kg fat) and fluid was consumed ad libitum. From −4 to −1 week, energy intake was reduced to 1300–1500 kcal, occurring by a reduction in protein intake to approximately 1 g/kg body mass. Training-wise, the first phase consisted weekly of two strength and conditioning sessions (e.g., weight training), eight combat-specific training sessions (e.g., grappling, striking, boxing, wrestling, MMA-specific) and two sparring sessions. During the second phase, only 1000 kcal per day was consumed, but protein intake was increased (0.5 g/kg CHO, 1.3 g/kg protein, 0.5 g/kg fat). The athlete also engaged in water loading where 8 L of water was consumed daily over a 4-day period prior to water intake being reduced to 0.25 L until the evening before weigh-in. Within the third phase, the athlete was subjected to a 20-h acute weight-cut period where no food or fluid was consumed while a series of continuous cycles of sweating were performed. Phase four consisted of rapid weight gain prior to the competitive event and phase five included two weeks of post-competition recovery. Since the main emphasis of the present work is RWL, we will focus mainly on the first three phases of the study. Body mass declined by 18.1% (80.2 to 65.7 kg) corresponding to changes of 4.4, 2.8 and 7.3 kg in phase 1, 2 and 3, respectively. After completing phase three, the athlete’s serum Cr values were 177 μmol/L. The authors concluded that the relative (almost a 50% increase) and absolute changes (53 μmol/L) in serum Cr levels during this final phase of the weight cut were consistent with acute kidney injury.

Fourteen male MMA fighters (23 ± 4 years; 1.76 ± 0.4 m; 76.8 ± 9.3 kg) volunteered to participate in study by Barley et al. [19] who sought to examine the influence of acute dehydration on physical performance and physiology in MMA athletes. Weight loss was prompted mainly through dehydration, where the RWL group took part in 3-h cycling at 60 W in 40 °C to induce 5% dehydration, while the control group underwent the same exercise protocol but in a thermoneutral environment (25 °C) exercise, followed by ad libitum fluid/food intake. Significant weight loss (*p* < 0.001) was detected in the RWL group (4.8 ± 0.8%). USG was significantly elevated from the baseline following RWL and was significantly greater than in the control group at 20 min (1.025 ± 0.01; *p* < 0.01) and 24 h post (1.022; *p* < 0.01) RWL.

## 4. Discussion

The aim of this study was to review the existing literature in order to examine the effects of RWL on kidney function in combat sport athletes. Monitoring of kidney function was reflected in observing Cr, BUN and USG values during or/and after RWL. Retrieved studies have demonstrated significantly increased Cr and BUN, and thus indicated acute kidney damage as a consequence of RWL. Increased values were also noted for USG, showing that combat sport athletes were dehydrated during or immediately after RWL. It is becoming increasingly recognized that acute kidney injury and chronic kidney disease are closely linked and likely promote one another [24]. However, USG values should be observed with caution, as many factors, such as weight reduction, training, urine metabolites, increased muscle mass and supplement consumption, may artificially increase urine concentrations, leading to false-positive findings [25].

It is important to outline that the included studies varied vastly in terms of research design, particularly in the number of days (or even weeks) permitted for the RWL procedure. For instance, studies by Karila et al. [15] and Cicioglu et al. [16] granted participants two to three weeks to reduce their weight, while Cannataro et al. [14] incorporated 3-day RWL in their study. Knowing that this fundamental aspect of research design was markedly different among included studies, it is very hard to make a comparison with respect to a degree of acute kidney damage. The literature defines RWL as a 5% body weight reduction in less than a week [12]. However, it remains a matter of debate whether a higher percentage of weight loss achieved over a longer period of time can still be considered as RWL. Moreover, some studies provided supplementation and supervised diet for their participants, while others did not. Likewise, some studies allowed participants to use self-selected means to induce RWL without reporting them to researchers, while others had to explicitly list patterns of RWL and even keep a food diary. These heterogenic properties of the included studies likely influenced the different outcomes found in the abovementioned studies. Indeed, there is no universally accepted definition of weight cycling but many possible variations on the same theme [26]. Still, the common feature between all RWL approaches is to maximize weight loss during the last couple of days prior to weigh-in (competition).

The current literature recognizes that dehydration is well known to be associated with acute renal dysfunction, but was originally considered reversible and to be associated with no long-term effects on the kidney function [27]. However, novel discoveries have led to recognition that even mild dehydration may be a risk factor in progression of all types of chronic kidney diseases [27]. Taking into consideration that some combat sport athletes engage in RWL up to 10 times annually [9], it seems reasonable that this frequently repeated cycle can certainly have effects on kidney function. In the context of combat sports, dehydration has been associated with a reduced plasma volume [28], which consequently leads to an increase in blood viscosity, which further reflects on cardiovascular efficiency [29] and increases the risk of acute cardiovascular issues. The aforementioned risks apply even when total water loss is modest [30]. Although modest, dehydration can impair both cognitive and [31,32] exercise performance [33,34], while increasing the physiological strain [35] at a given intensity of exercise. It should be stressed, that the studies included within the present review looked at the transient effect of RWL and intentional dehydration as its key feature. To our knowledge, no studies with a substantial follow-up period have elaborated on kidney damage as a consequence of RWL practice. The magnitude of the RWL effect is likely dependent on a multitude of factors, such as frequency of weight cycling events per year, the extent of weight loss, level of experience, training status and subsequent recovery. Based on the current findings, we hypothesize that the consequences of such practices certainly resemble in the kidney function chronically, although more studies are needed on this topic. Kasper et al. [21] suggested that MMA athletes may be particularly sensitive to long-term kidney complications, as several MMA athletes have retired from the sport citing kidney disease as one of the main factors. In addition, several studies reveal that the metabolic consequences of weight cycling certainly occur in athletes who perform RWL and can have lifetime implications [26,36].

Based on these and other hazards of RWL eloquently described in the existing literature, it is for a good reason that experts in the field of combat sports are calling for RWL to be banned from combat sports [10]. According to Artioli et al. [10], RWL fulfills the World Anti-Doping Agency’s criteria to be banned since it has the potential to enhance sport performance, it represents a health risk to the athlete and it violates the spirit of the sport. Ethical consequences of RWL are often overlooked, yet in the environment where it is widely accepted to drop one or two weight classes, all athletes may feel forced to follow this tendency to avoid unfair competition against a bigger and stronger opponent, resulting in an unfortunate cascade effect [10].

Gradual weight loss has been proposed as an alternative strategy to the current approach of RWL [5], whereby weight loss would be achieved at the slower rate and ideally from fat deposits. Referring to the existing situation of RWL in combat sports, the American College of Sports Medicine stated that key methods for weight loss (e.g., increased exercise, caloric deficit, fasting and various dehydration methods) primarily affect body water, glycogen content and lean body mass rather than targeting fat loss [7]. Using sophisticated body composition measurement devices to monitor athletes’ weight oscillations during RWL would allow us to determine what type of bodily tissues are being lost, whereby training and diet can be adjusted accordingly to initiate fat loss.

Hoffman et al. [37] emphasized that an adequate intake of high-quality carbohydrates must be ensured as this macronutrient is a primary fuel used by combat sport athletes during training and competition. Of particular importance for combat sport athletes during RWL or gradual weight loss is sufficient protein intake, as during prolonged situations of low energy intake, elevated protein consumption appears to protect lean tissue and prevent or minimize the catabolic impact of insufficient caloric diets [38].

Personal efforts precluding RWL must be led by organizational legislation [39] that discourages RWL and emphasizes fairness that benefits the sport. A mandatory hydration test that accompanies weigh-in, minimal competitive weight determination that will be screened quarterly and narrowing the time-frame between weigh-in and competition are some of the strategies advocated by the experts to prevent RWL.

## 5. Conclusions

Rapid weight loss is associated with significant acute kidney damage in combat sport athletes. It seems that elevated biomarkers of kidney function can be primarily attributed to intentional dehydration, which is the culprit of rapid weight loss.

## Figures and Tables

**Table 1:** Effects of rapid weight loss on kidney function in combat sport athletes.

Study	Sport	Sample	RWL Period	Mean Weight Loss (%)	*p*	
Blood Urea Nitrogen						
Cannataro et al. [14]	Muay Thai	*N* = 21 M = 13 F = 8	3 days	4.1	M	F
					↑*	↑*
Cicioglu et al. [16]	Wrestling	*N* = 24(M)	1–2 weeks	5.7	↑**	
Ozkan and Cicioglu [23]	Wrestling	*N* = 69(M)	1–7 days	4.5 ± 1.8	↑*	
Creatinine						
Karila et al. [15]	Wrestling	*N* = 18(M)	2–3 weeks	8.2 ± 2.3	↑**	
Kasper et al. [21]	MMA	*N* = 1(M)	8 weeks	14.5	↑**	
Drid et al. [22]	Judo	*N* = 8(M)	7 days	6	↑*	
Yang et al. [18]	Taekwondo	*N* = 10(M)	4 days	5	↑*	
Urine Specific Gravity						
Timpmann et al. [17]	Wrestling	*N* = 16(M)	3 days	5	↑**	
Barley et al. [19]	MMA	*N* = 14(M)	3 h	4.8 ± 0.8	↑*	
Jetton et al. [20]	MMA	*N* = 40 M = 38 F = 2	~1 day	4.4	↓**	

RWL period—rapid weight loss period; MMA—mixed martial arts; *N*—number of participants; M—males; F—females; ↑*—significant increase, *p* ≤ 0.05; ↑**—significant increase, *p* ≤ 0.001; ↓**—significant decrease, *p* ≤ 0.001.

## Data Availability

Available upon reasonable request.
